# Crystal structure, Hirshfeld surface analysis and DFT study of *N*-(2-amino-5-methyl­phen­yl)-2-(5-methyl-1*H*-pyrazol-3-yl)acetamide

**DOI:** 10.1107/S205698902100503X

**Published:** 2021-05-14

**Authors:** Gamal Al Ati, Karim Chkirate, Joel T. Mague, Nadeem Abad, Redouane Achour, El Mokhtar Essassi

**Affiliations:** a Laboratory of Heterocyclic Organic Chemistry URAC 21, Pharmacochemistry Competence Center, Av. Ibn Battouta, BP 1014, Faculty of Sciences, Mohammed V University, Rabat, Morocco; b Department of Chemistry, Tulane University, New Orleans, LA 70118, USA; c Department of Biochemistry, Faculty of Education & Science, Al-Baydha University, Yemen

**Keywords:** crystal structure, pyrazolylacetamide, hydrogen bond

## Abstract

The title mol­ecule, C_13_H_16_N_4_O, adopts an angular conformation. In the crystal a layer structure is generated by N—H⋯O and N—H⋯N hydrogen bonds together with C—H⋯π(ring) inter­actions. Hirshfeld surface analysis indicates that the most important contributions to the crystal packing are from H⋯H (53.8%), H⋯C/C⋯H (21.7%), H⋯N/N⋯H (13.6%), and H⋯O/O⋯H (10.8%) inter­actions. The optimized structure calculated using density functional theory (DFT) at the B3LYP/ 6–311 G(d,p) level is compared with the experimentally determined structure in the solid state. The calculated HOMO–LUMO energy gap is 5.0452 eV.

## Abstract 2

The title mol­ecule adopts an angular conformation. In the crystal, N—H⋯O and N—H⋯N hydrogen bonds together with C—H⋯π(ring) inter­actions form chains extending along the *a*-axis direction. Additional N—H⋯O hydrogen bonds link the chains into layers parallel to (100).

## Chemical context   

Nitro­gen-based structures have attracted more attention in recent years because of their inter­esting properties in structural and inorganic chemistry (Lahmidi *et al.*, 2018 ▸; Chkirate *et al.*, 2020*a* ▸; Taia *et al.*, 2020 ▸; Al Ati *et al.*, 2021 ▸). The pyrazolyl-acetamide family is important in medicinal chemistry because of the wide range of pharmacological applications (Deprez-Poulain *et al.*, 2011 ▸) such as anti-inflammatory (Sunder *et al.*, 2013 ▸), anti­microbial and anti­cancer (Jitender Dev *et al.*, 2017 ▸) and as an anti-amoebic agent (Shukla *et al.*, 2020 ▸). They also have anti­oxidant activity (Chkirate *et al.*, 2019*a* ▸) and have been biologically evaluated (Yan *et al.*, 2021 ▸). Given the wide range of therapeutic applications for such compounds, and in a continuation of the work already carried out for the synthesis of compounds resulting from 1,5-benzodiazepine (Chkirate *et al.*, 2001 ▸, 2018 ▸, 2019*b* ▸, 2020*b* ▸, 2021 ▸; Idrissi *et al.*, 2021 ▸) a similar approach gave the title compound, *N*-(2-amino-5-methyl­phen­yl)-2-(5-methyl-1*H*-pyrazol-3-yl)acetamide, (I). Besides the synthesis, we also report the mol­ecular and crystal structures along with a Hirshfeld surface analysis and a density functional theory computational calculation carried out at the B3LYP/6–311 G(d,p) level.

## Structural commentary   

**Figure 1:**
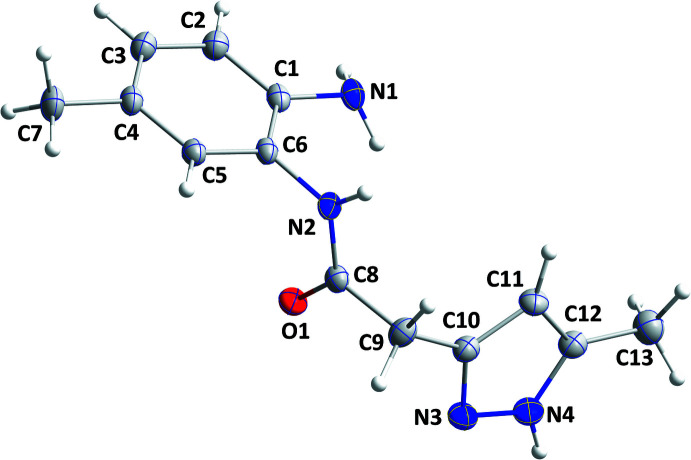
Mol­ecular structure of the title compound with the labelling scheme. The ellipsoids are drawn at the 50% probability level.

The N2/C8/C9/O1 portion of the title mol­ecule is planar (r.m.s. deviation = 0.0013 Å) with the mean planes of the C1–C6 and N3/N4/C10–C12 rings inclined to the above plane by 86.56 (6) and 72.84 (7)°, respectively, giving the mol­ecule an angular shape (Fig. 1 ▸). Bond distances and angles are as expected for the given formulation.
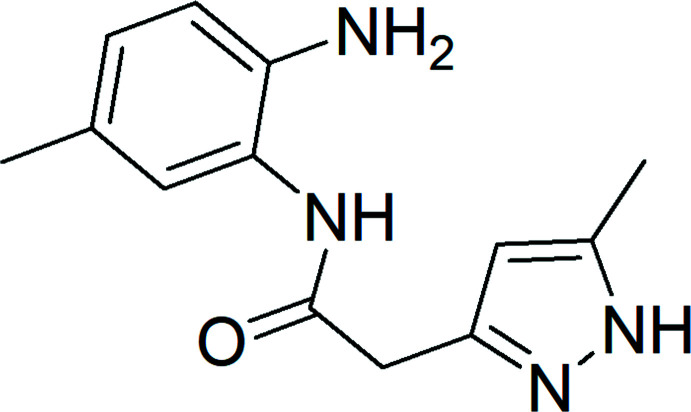


## Supra­molecular features   

**Figure 2:**
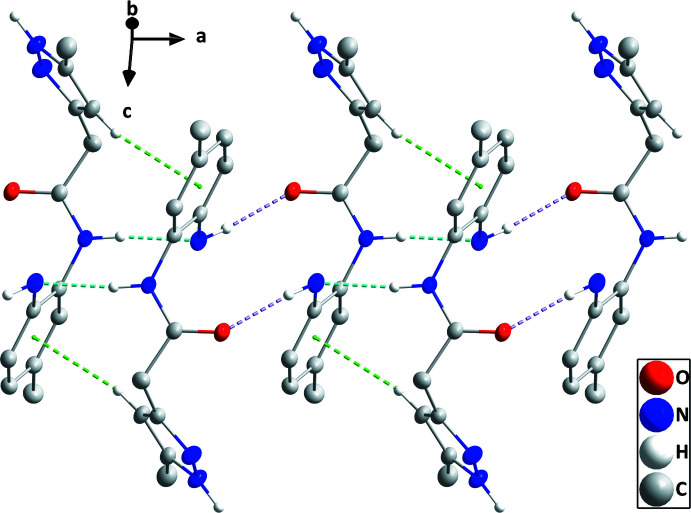
A portion of one chain projected onto (011) with N—H⋯O and N—H⋯N hydrogen bonds depicted, respectively, by light-purple and light-blue dashed lines. The C—H⋯π(ring) inter­actions are depicted by green dashed lines. Hydrogen atoms not involved in inter­actions have been omitted for clarity.

**Figure 3:**
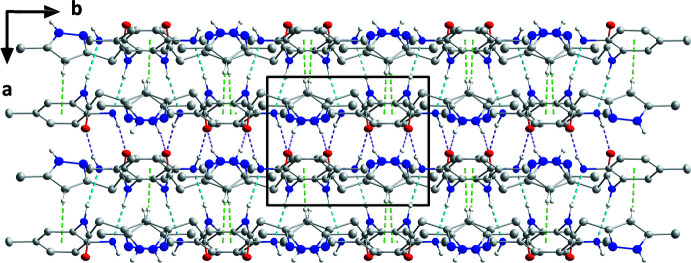
Packing arrangement viewed along the *c*-axis direction of the main isomer with inter­molecular inter­actions shown as in Fig. 2 ▸.

In the crystal, inversion-related pairs of N1—H1*B*⋯O1, and N2—H2*A*⋯N1 hydrogen bonds, together with C11—H11⋯*Cg*2 inter­actions (Table 1 ▸) form chains of mol­ecules extending along the *a*-axis direction (Fig. 2 ▸). The chains are connected into layers parallel to (100) by N4—H4⋯O1 hydrogen bonds (Table 1 ▸ and Fig. 3 ▸). Inter­molecular inter­actions viewed down the *c* axis are shown in Fig. 3 ▸.

## Hirshfeld surface analysis   

**Figure 4:**
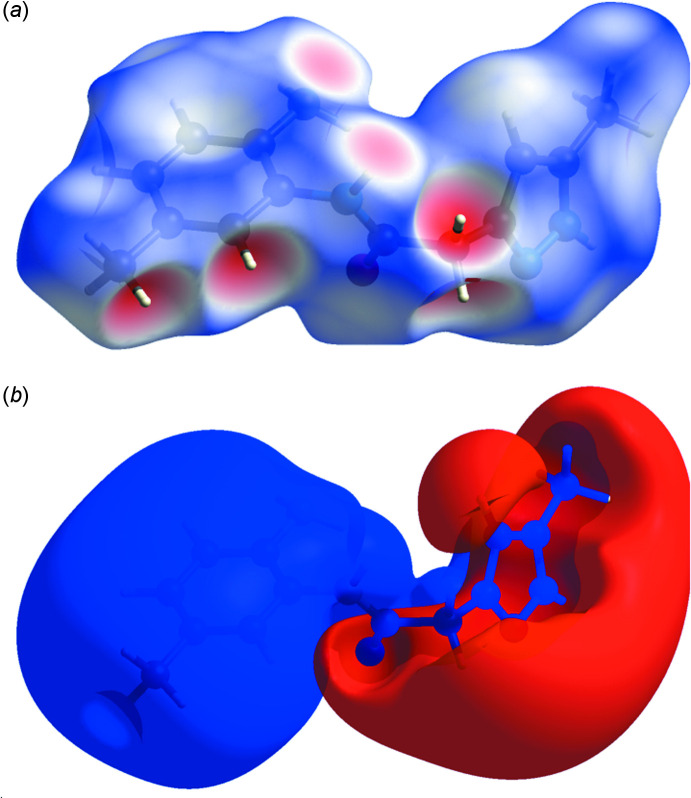
(*a*) View of the three-dimensional Hirshfeld surface of the title compound, plotted over *d*_norm_ in the range of −0.6149 to 1.3177 a.u. (*b*) View of the three-dimensional Hirshfeld surface of the title compound plotted over the electrostatic potential energy in the range −0.0500 to 0.0500 a.u. using the STO-3 G basis set at the Hartree–Fock level of theory.

**Figure 5:**
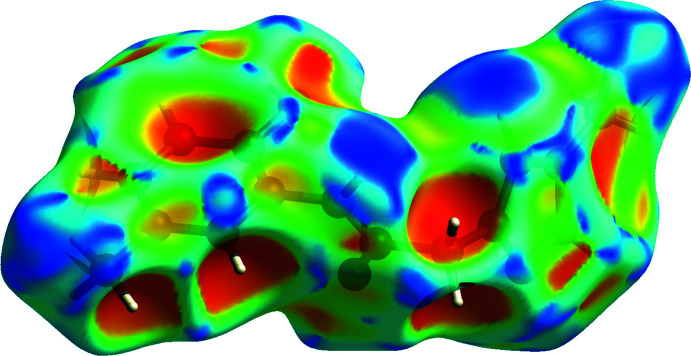
Hirshfeld surface of the title compound plotted over shape-index.

The *CrystalExplorer* program (Turner *et al.*, 2017 ▸) was used to investigate and visualize further the inter­molecular inter­actions of (I). The Hirshfeld surface plotted over *d*_norm_ in the range −0.6149 to 1.3177 a.u. is shown in Fig. 4 ▸ *a*. The electrostatic potential calculated using the STO-3G basis set at the Hartree–Fock level of theory and mapped on the Hirshfeld surface over the range ±0.05 a.u. clearly shows the positions of close inter­molecular contacts in the compound (Fig. 4 ▸ *b*). The positive electrostatic potential (blue region) over the surface indicates hydrogen-donor potential, whereas the hydrogen-bond acceptors are represented by negative electrostatic potential (red region). The shape-index (Fig. 5 ▸) generated in the range −1 to 1 Å reveals that there are no significant π–π inter­actions, normally indicated by adjacent red and blue triangles.

**Figure 6:**
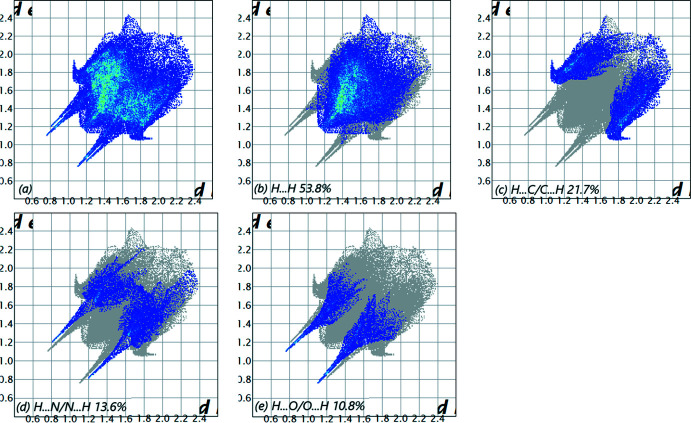
The full two-dimensional fingerprint plots for the title compound, showing (*a*) all inter­actions, and those delineated into (*b*) H⋯H, (*c*) H⋯C/C⋯H, (*d*) H⋯N/N⋯H and (*e*) H⋯O/O⋯H inter­actions. The *d*_i_ and *d*_e_ values are the closest inter­nal and external distances (in Å) from given points on the Hirshfeld surface.

The overall two-dimensional fingerprint plot (McKinnon *et al.*, 2007 ▸) is shown in Fig. 6 ▸ *a*, while those delineated into H⋯H, H⋯C/C⋯H, H⋯N/N⋯H and H⋯O/O⋯H contacts are illustrated in Fig. 6 ▸ *b*–*e*, respectively, together with their relative contributions to the Hirshfeld surface (HS). The most important inter­action is H⋯H, contributing 53.8% to the overall crystal packing, which is reflected in Fig. 6 ▸ *b* as widely scattered points of high density due to the large hydrogen content of the mol­ecule, with the tip at *d*_e_ = *d*_i_ = 1.18 Å. In the presence of C—H inter­actions, the pair of characteristic wings in the fingerprint plot delineated into H⋯C/C⋯H contacts (21.7% contribution to the HS), Fig. 6 ▸ *c*, has the tips at *d*_e_ + *d*_i_ = 2.76 Å. The pair of scattered points of spikes in the fingerprint plot delineated into H⋯N/N⋯H, Fig. 6 ▸ *d* (13.6%), have the tips at *d*_e_ + *d*_i_ = 2.01 Å. Finally, the H⋯O/O⋯H contacts, Fig. 6 ▸ *e*, make only a 10.8% contribution to the HS and have a low-density distribution of points.

## Density functional theory calculations   

**Figure 7:**
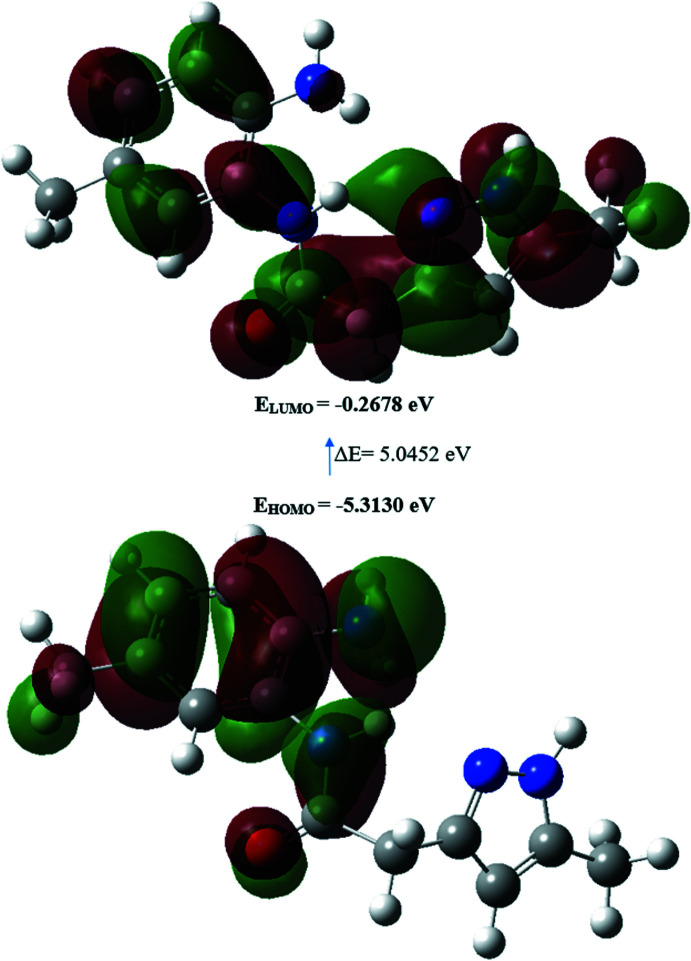
The energy band gap of *N*-(2-amino-5-methyl­phen­yl)-2-(5-methyl-1*H*-pyrazol-3-yl)acetamide.

The structure in the gas phase of the title compound was optimized by means of density functional theory. The density functional theory calculation was performed by the hybrid B3LYP method and the 6–311 G(d,p) basis-set, which is based on Becke’s model (Becke, 1993 ▸) and considers a mixture of the exact (Hartree–Fock) and density functional theory exchange utilizing the B3 functional, together with the LYP correlation functional (Lee *et al.*, 1988 ▸). After obtaining the converged geometry, the harmonic vibrational frequencies were calculated at the same theoretical level to confirm that the number of imaginary frequencies is zero for the stationary point. Both the geometry optimization and harmonic vibrational frequency analysis of the title compound were done with the *Gaussian 09* program (Frisch *et al.*, 2009 ▸). Theoretical and experimental results related to bond lengths and angles are in good agreement and are summarized in Table 2 ▸. Calculated numerical values for the title compound including electronegativity (*χ*), hardness (*η*), ionization potential (*I*), dipole moment (*μ*), electron affinity (*A*), electrophilicity (*ω*) and softness (*σ*) are collated in Table 3 ▸. The electron transition from the highest occupied mol­ecular orbital (HOMO) to the lowest unoccupied mol­ecular orbital (LUMO) energy level is shown in Fig. 7 ▸. The HOMO and LUMO are localized in the plane extending over the whole *N*-(2-amino-5-methyl­phen­yl)-2-(5-methyl-1*H*-pyrazol-3-yl)acetamide system. The energy band gap [*ΔE* = *E_LUMO_* − *E_HOMO_*] of the mol­ecule is 5.0452 eV, and the frontier mol­ecular orbital energies, *E_HOMO_* and *E_LUMO_*, are −5.3130 and −0.2678 eV, respectively.

## Database survey   

**Figure 8:**
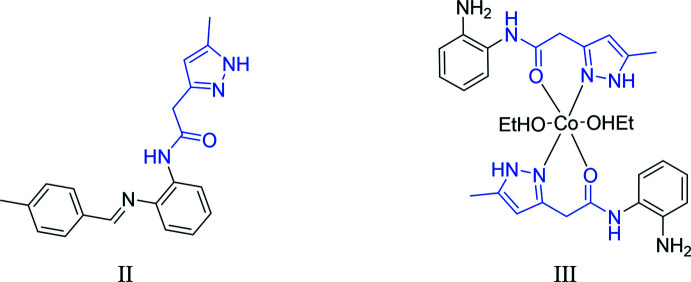
Structures similar to (I): (II) (CSD refcode XITFUE) and (III) (CSD refcode YODZEZ) obtained in the database search. The search fragment is indicated in blue.

A search of the Cambridge Structural Database (CSD version 5.40, updated March 2020; Groom *et al.*, 2016 ▸) with the 2-(5-methyl-1*H*-pyrazol-3-yl)acetamide fragment yielded multiple matches. Of these, two had an *N*-(2-amino­phen­yl) substituent comparable to (I) and they are shown in Fig. 8 ▸. The first compound (II) (refcode XITFUE; Chkirate *et al.*, 2019*c* ▸) carries *N*-(2-{[(4-methyl­phen­yl)methyl­idene]amino}­phen­yl) on nitro­gen 2. The second one (III) (refcode YODZEZ; Chkirate *et al.*, 2019*a* ▸) carries *N*-(2-amino­phen­yl) on nitro­gen 2. The pyrazole ring (N3/N4/C10–C12) in XITFUE is inclined to the C1–C6 benzene ring by 70.83 (8)°. In YODZEZ, the dihedral angle between the mean planes of the 2-amino­phenyl and pyrazolyl rings is 65.63 (8)°. In (I), the N2/C8/C9/O1 fragment is planar (r.m.s. deviation = 0.0013 Å) with the mean planes of the C1–C6 and N3/N4/C10–C12 rings inclined to the above plane by 86.56 (6) and 72.84 (7)°, respectively, which is approximately the same as in XITFUE, but less tilted than in YODZEZ.

## Synthesis and crystallization   

2 g (9.3 mmol) of (*Z*)-4-(2-oxo­propyl­idene)-1,5-benzodiazepin-2-one and a stoichiometric amount of hydrazine were refluxed in ethanol (40 mL) for 2 h. After concentration of the solvent volume to 20 mL, the solution was allowed to stand; the precipitate formed was filtered off and then recrystallized in ethanol. Single crystals were obtained after recrystallization from methanol in the presence of MnCl_2_·4H_2_O, which was left at room temperature for 72 h. Yield: 70%.

## Refinement   

Crystal data, data collection and structure refinement details are summarized in Table 4 ▸. H atoms were included as riding contributions in idealized positions (N—H = 0.88–0.91 Å, C—H = 0.95–0.99 Å) with isotropic displacement parameters 1.2–1.5 times those of the attached atoms. Residual density observed after the initial refinement converged was identified as an isomer of the primary mol­ecule having the C7 methyl group attached to C3 instead of to C4 and with a refined occupancy of 5%. The final model was generated with a combination of rigid group and restrained refinement to make the minor component have a comparable geometry to that of the major component.

## Supplementary Material

Crystal structure: contains datablock(s) global, I. DOI: 10.1107/S205698902100503X/dj2025sup1.cif

Structure factors: contains datablock(s) I. DOI: 10.1107/S205698902100503X/dj2025Isup5.hkl

Supporting information file. DOI: 10.1107/S205698902100503X/dj2025Isup3.cml

CCDC reference: 2083102

Additional supporting information:  crystallographic information; 3D view; checkCIF report

## Figures and Tables

**Table 1:** Hydrogen-bond geometry (Å, °)

*D*—H⋯*A*	*D*—H	H⋯*A*	*D*⋯*A*	*D*—H⋯*A*
N1—H1*B*⋯O1^i^	0.91	2.13	3.0284 (19)	171
N2—H2*A*⋯N1^ii^	0.91	2.14	3.0354 (17)	170
C2—H2⋯O1^i^	0.95	2.62	3.334 (2)	132
N4—H4⋯O1^iii^	0.91 (1)	1.99 (1)	2.8625 (17)	163 (2)

Symmetry codes: (i) -x+1, -y+1, -z+1; (ii) -x+2, -y+1, -z+1; (iii) -x+1, y+{\script{1\over 2}}, -z+{\script{1\over 2}}.

**Table 2:** Comparison of selected (X-ray and density functional theory) bond lengths and angles (Å, °)

	X-ray	B3LYP/6–311G(d,p)
N1—C1	1.4112 (17)	1.4114
N2—C6	1.4347 (17)	1.4139
N2—C8	1.3471 (17)	1.3692
O1—C8	1.2376 (16)	1.2179
N3—C10	1.3425 (18)	1.3316
N3—N4	1.3635 (19)	1.3524
N4—C12	1.3534 (19)	1.3598
C8—C9	1.5119 (18)	1.5409
C9—C10	1.496 (2)	1.5007
		
C2—C1—N1	121.12 (12)	122.0542
C6—C1—N1	120.68 (12)	119.3119
C1—C6—N2	119.89 (11)	116.726
C5—C6—N2	119.46 (12)	123.4969
O1—C8—N2	122.20 (12)	125.0222
N2—C8—C9	116.22 (11)	114.6561
O1—C8—C9	121.58 (12)	120.2798
N3—C10—C9	119.95 (13)	120.7841
N3—C10—C11	111.17 (12)	110.8968
C10—N3—N4	104.22 (12)	104.754
C12—N4—N3	112.76 (12)	113.2928
N4—C12—C11	106.48 (13)	105.3557
N4—C12—C13	122.33 (14)	122.8603

**Table 3:** Calculated energies

Mol­ecular Energy	Compound (I)
Total Energy *TE* (eV)	−21754.8403
*E*_HOMO_ (eV)	−5.3130
*E*_LUMO_ (eV)	−0.2678
Gap, *ΔE* (eV)	5.0452
Dipole moment, *μ* (Debye)	6.7706
Ionization potential, *I* (eV)	5.3130
Electron affinity, *A*	0.2678
Electronegativity, *χ*	2.7904
Hardness, *η*	2.5226
Electrophilicity, index *ω*	1.5433
Softness, *σ*	0.3964
Fraction of electron transferred, *ΔN*	0.8344

**Table 4:** Experimental details

Crystal data	
Chemical formula	C_13_H_16_N_4_O
*M*_r_	244.30
Crystal system, space group	Monoclinic, *P*2_1_/*c*
Temperature (K)	150
*a*, *b*, *c* (Å)	7.1271 (3), 8.9295 (3), 19.2508 (7)
β (°)	94.683 (1)
*V* (Å^3^)	1221.06 (8)
*Z*	4
Radiation type	Mo *K*α
μ (mm^−1^)	0.09
Crystal size (mm)	0.37 × 0.26 × 0.16

Data collection	
Diffractometer	Bruker D8 QUEST PHOTON 3 diffractometer
Absorption correction	Numerical (*SADABS*; Krause *et al.*, 2015 ▸)
*T*_min_, *T*_max_	0.93, 0.99
No. of measured, independent and observed [*I* > 2σ(*I*)] reflections	66426, 5120, 4794
*R*_int_	0.028
(sin θ/λ)_max_ (Å^−1^)	0.794

Refinement	
*R*[*F* ^2^ > 2σ(*F* ^2^)], *wR*(*F* ^2^), *S*	0.067, 0.164, 1.23
No. of reflections	5120
No. of parameters	212
No. of restraints	32
H-atom treatment	H atoms treated by a mixture of independent and constrained refinement
Δρ_max_, Δρ_min_ (e Å^−3^)	0.44, −0.33

Computer programs: *APEX3* and *SAINT* (Bruker, 2020 ▸), *SHELXT* (Sheldrick, 2015*a* ▸), *SHELXL2018/1* (Sheldrick, 2015*b* ▸), *DIAMOND* (Brandenburg & Putz, 2012 ▸) and *SHELXTL* (Sheldrick, 2008 ▸).

## supplementary crystallographic information

### Crystal data

**Table:**

C_13_H_16_N_4_O	*F*(000) = 520
*M**_r_* = 244.30	*D*_x_ = 1.329 Mg m^−^^3^
Monoclinic, *P*2_1_/*c*	Mo *K*α radiation, λ = 0.71073 Å
*a* = 7.1271 (3) Å	Cell parameters from 9878 reflections
*b* = 8.9295 (3) Å	θ = 2.5–34.4°
*c* = 19.2508 (7) Å	µ = 0.09 mm^−^^1^
β = 94.683 (1)°	*T* = 150 K
*V* = 1221.06 (8) Å^3^	Parallelepiped, colourless
*Z* = 4	0.37 × 0.26 × 0.16 mm

### Data collection

**Table:**

Bruker D8 QUEST PHOTON 3 diffractometer	5120 independent reflections
Radiation source: fine-focus sealed tube	4794 reflections with *I* > 2σ(*I*)
Graphite monochromator	*R*_int_ = 0.028
Detector resolution: 7.3910 pixels mm^-1^	θ_max_ = 34.4°, θ_min_ = 2.5°
φ and ω scans	*h* = −11→11
Absorption correction: numerical (*SADABS*; Krause *et al.*, 2015)	*k* = −14→14
*T*_min_ = 0.93, *T*_max_ = 0.99	*l* = −30→30
66426 measured reflections	

### Refinement

**Table:**

Refinement on *F*^2^	Primary atom site location: dual
Least-squares matrix: full	Secondary atom site location: difference Fourier map
*R*[*F*^2^ > 2σ(*F*^2^)] = 0.067	Hydrogen site location: mixed
*wR*(*F*^2^) = 0.164	H atoms treated by a mixture of independent and constrained refinement
*S* = 1.23	*w* = 1/[σ^2^(*F*_o_^2^) + (0.0465*P*)^2^ + 0.8885*P*] where *P* = (*F*_o_^2^ + 2*F*_c_^2^)/3
5120 reflections	(Δ/σ)_max_ = 0.007
212 parameters	Δρ_max_ = 0.44 e Å^−^^3^
32 restraints	Δρ_min_ = −0.33 e Å^−^^3^

### Special details

**Table:**

Experimental. The diffraction data were obtained from 9 sets of frames, each of width 0.5° in ω or φ, collected with scan parameters determined by the "strategy" routine in *APEX3*. The scan time was 15 sec/frame.
Geometry. All esds (except the esd in the dihedral angle between two l.s. planes) are estimated using the full covariance matrix. The cell esds are taken into account individually in the estimation of esds in distances, angles and torsion angles; correlations between esds in cell parameters are only used when they are defined by crystal symmetry. An approximate (isotropic) treatment of cell esds is used for estimating esds involving l.s. planes.
Refinement. Refinement of F^2^ against ALL reflections. The weighted R-factor wR and goodness of fit S are based on F^2^, conventional R-factors R are based on F, with F set to zero for negative F^2^. The threshold expression of F^2^ > 2sigma(F^2^) is used only for calculating R-factors(gt) etc. and is not relevant to the choice of reflections for refinement. R-factors based on F^2^ are statistically about twice as large as those based on F, and R- factors based on ALL data will be even larger. H-atoms were included as riding contributions in idealized positions with isotropic displacement parameters 1.2 - 1.5 times those of the attached atoms. Residual density observed after the initial refinement converged was identified as an isomer of the primary molecule having the C7 methyl group attached to C3 instead of to C4 and with a refined occupancy of 5%. The final model was generated with a combination of rigid group and restrained refinement to make the minor component have a comparable geometry to that of the major component.

### Fractional atomic coordinates and isotropic or equivalent isotropic displacement parameters (Å^2^)

**Table:**

	*x*	*y*	*z*	*U*_iso_*/*U*_eq_	Occ. (<1)
O1	0.59624 (16)	0.37274 (17)	0.37033 (6)	0.0232 (2)	0.9480 (17)
N1	0.71759 (18)	0.54183 (13)	0.54033 (7)	0.0217 (2)	0.9480 (17)
H1A	0.705685	0.575800	0.495619	0.032*	0.9480 (17)
H1B	0.625376	0.578103	0.565705	0.032*	0.9480 (17)
N2	0.87261 (16)	0.36438 (14)	0.43708 (6)	0.0194 (2)	0.9480 (17)
H2A	0.997675	0.386010	0.438829	0.029*	0.9480 (17)
O1A	0.611 (2)	0.392 (4)	0.3551 (15)	0.0232 (2)	0.0520 (17)
N1A	0.726 (2)	0.5270 (12)	0.5246 (10)	0.0217 (2)	0.0520 (17)
H1C	0.714407	0.560992	0.479901	0.032*	0.0520 (17)
H1D	0.634098	0.563294	0.549991	0.032*	0.0520 (17)
N2A	0.892 (3)	0.379 (3)	0.4164 (9)	0.0194 (2)	0.0520 (17)
H2C	1.010632	0.408561	0.419618	0.023*	0.0520 (17)
C1	0.71306 (17)	0.38417 (14)	0.54490 (7)	0.0183 (2)	0.9480 (17)
C2	0.63639 (19)	0.31229 (16)	0.60084 (8)	0.0211 (2)	0.9480 (17)
H2	0.581442	0.370326	0.635231	0.025*	0.9480 (17)
C3	0.63985 (19)	0.15736 (16)	0.60656 (8)	0.0217 (2)	0.9480 (17)
H3	0.588038	0.111015	0.645085	0.026*	0.9480 (17)
C4	0.71811 (19)	0.06826 (15)	0.55670 (7)	0.0201 (2)	0.9480 (17)
C5	0.79467 (18)	0.13954 (15)	0.50122 (7)	0.0190 (2)	0.9480 (17)
H5	0.848761	0.081079	0.466773	0.023*	0.9480 (17)
C6	0.79337 (17)	0.29531 (14)	0.49529 (7)	0.0173 (2)	0.9480 (17)
C7	0.7163 (2)	−0.10021 (16)	0.56184 (9)	0.0267 (3)	0.9480 (17)
H7A	0.762818	−0.143428	0.519718	0.040*	0.9480 (17)
H7B	0.587355	−0.134775	0.566403	0.040*	0.9480 (17)
H7C	0.797458	−0.131927	0.602734	0.040*	0.9480 (17)
C8	0.76818 (18)	0.39484 (15)	0.37725 (7)	0.0181 (2)	0.9480 (17)
C9	0.87268 (19)	0.45874 (17)	0.31874 (7)	0.0233 (3)	0.9480 (17)
H9A	1.009431	0.458403	0.332792	0.028*	0.9480 (17)
H9B	0.850612	0.394239	0.277043	0.028*	0.9480 (17)
C1A	0.7392 (15)	0.3693 (12)	0.5253 (6)	0.0183 (2)	0.0520 (17)
C2A	0.664 (2)	0.2879 (16)	0.5780 (7)	0.0211 (2)	0.0520 (17)
H2B	0.604271	0.338398	0.613539	0.025*	0.0520 (17)
C3A	0.677 (2)	0.1325 (16)	0.5786 (8)	0.0217 (2)	0.0520 (17)
C4A	0.764 (2)	0.0586 (12)	0.5266 (9)	0.0201 (2)	0.0520 (17)
H4A	0.773122	−0.047555	0.527024	0.024*	0.0520 (17)
C5A	0.839 (2)	0.1400 (14)	0.4739 (8)	0.0190 (2)	0.0520 (17)
H5A	0.899373	0.089494	0.438300	0.023*	0.0520 (17)
C6A	0.827 (2)	0.2954 (14)	0.4732 (7)	0.0173 (2)	0.0520 (17)
C7A	0.603 (4)	0.043 (3)	0.6369 (11)	0.0267 (3)	0.0520 (17)
H7D	0.545542	0.110860	0.669177	0.040*	0.0520 (17)
H7E	0.508277	−0.028421	0.617575	0.040*	0.0520 (17)
H7F	0.706943	−0.011442	0.661987	0.040*	0.0520 (17)
C8A	0.783 (2)	0.415 (3)	0.3584 (9)	0.0181 (2)	0.0520 (17)
C9A	0.890 (3)	0.487 (2)	0.3025 (7)	0.0233 (3)	0.0520 (17)
H9C	1.025101	0.495079	0.318091	0.028*	0.0520 (17)
H9D	0.876172	0.427022	0.259173	0.028*	0.0520 (17)
N3	0.67894 (16)	0.63909 (15)	0.24804 (6)	0.0255 (3)	0.9480 (17)
N4	0.65868 (16)	0.79091 (15)	0.24611 (6)	0.0252 (3)	0.9480 (17)
H4	0.574 (3)	0.834 (3)	0.2145 (10)	0.036 (6)*	0.9480 (17)
C10	0.81137 (15)	0.61509 (16)	0.30054 (6)	0.0208 (2)	0.9480 (17)
C11	0.87425 (16)	0.75120 (17)	0.33163 (7)	0.0218 (2)	0.9480 (17)
H11	0.966736	0.764031	0.369572	0.026*	0.9480 (17)
C12	0.77310 (17)	0.86140 (17)	0.29529 (6)	0.0219 (3)	0.9480 (17)
C13	0.7758 (2)	1.0282 (2)	0.30308 (9)	0.0301 (3)	0.9480 (17)
H13A	0.898183	1.059745	0.325150	0.045*	0.9480 (17)
H13B	0.675980	1.059071	0.332152	0.045*	0.9480 (17)
H13C	0.754972	1.074905	0.257033	0.045*	0.9480 (17)
N3A	0.6378 (14)	0.668 (2)	0.2606 (3)	0.0255 (3)	0.0520 (17)
N4A	0.6309 (16)	0.819 (2)	0.2618 (3)	0.0252 (3)	0.0520 (17)
H4B	0.530526	0.868604	0.244696	0.036 (6)*	0.0520 (17)
C10A	0.8062 (14)	0.639 (2)	0.2901 (3)	0.0208 (2)	0.0520 (17)
C11A	0.900 (2)	0.773 (2)	0.3088 (5)	0.0218 (2)	0.0520 (17)
H11B	1.023731	0.780410	0.330902	0.026*	0.0520 (17)
C12A	0.783 (3)	0.891 (2)	0.2898 (6)	0.0219 (3)	0.0520 (17)
C13A	0.809 (4)	1.053 (3)	0.2971 (9)	0.0301 (3)	0.0520 (17)
H13D	0.934950	1.073535	0.319707	0.045*	0.0520 (17)
H13E	0.797247	1.099750	0.250883	0.045*	0.0520 (17)
H13F	0.713733	1.094159	0.325523	0.045*	0.0520 (17)

### Atomic displacement parameters (Å^2^)

**Table:**

	*U*^11^	*U*^22^	*U*^33^	*U*^12^	*U*^13^	*U*^23^
O1	0.0175 (4)	0.0273 (6)	0.0240 (6)	−0.0023 (4)	−0.0030 (4)	0.0038 (5)
N1	0.0196 (5)	0.0157 (5)	0.0292 (6)	0.0003 (4)	−0.0008 (4)	0.0004 (4)
N2	0.0152 (4)	0.0210 (5)	0.0216 (5)	−0.0027 (4)	−0.0012 (4)	0.0056 (4)
O1A	0.0175 (4)	0.0273 (6)	0.0240 (6)	−0.0023 (4)	−0.0030 (4)	0.0038 (5)
N1A	0.0196 (5)	0.0157 (5)	0.0292 (6)	0.0003 (4)	−0.0008 (4)	0.0004 (4)
N2A	0.0152 (4)	0.0210 (5)	0.0216 (5)	−0.0027 (4)	−0.0012 (4)	0.0056 (4)
C1	0.0140 (5)	0.0165 (5)	0.0237 (6)	−0.0001 (4)	−0.0026 (4)	0.0018 (4)
C2	0.0183 (5)	0.0204 (6)	0.0245 (6)	0.0016 (4)	0.0020 (4)	0.0013 (5)
C3	0.0192 (5)	0.0217 (6)	0.0244 (6)	−0.0005 (4)	0.0029 (4)	0.0049 (5)
C4	0.0185 (5)	0.0165 (5)	0.0250 (6)	−0.0017 (4)	−0.0003 (4)	0.0042 (4)
C5	0.0184 (5)	0.0170 (5)	0.0211 (5)	−0.0004 (4)	−0.0003 (4)	0.0012 (4)
C6	0.0153 (5)	0.0163 (5)	0.0200 (5)	−0.0016 (4)	−0.0012 (4)	0.0032 (4)
C7	0.0276 (6)	0.0175 (6)	0.0350 (7)	−0.0023 (5)	0.0027 (5)	0.0061 (5)
C8	0.0183 (5)	0.0153 (5)	0.0204 (6)	0.0008 (4)	0.0000 (4)	0.0021 (4)
C9	0.0233 (6)	0.0237 (6)	0.0233 (6)	0.0026 (5)	0.0052 (5)	0.0059 (5)
C1A	0.0140 (5)	0.0165 (5)	0.0237 (6)	−0.0001 (4)	−0.0026 (4)	0.0018 (4)
C2A	0.0183 (5)	0.0204 (6)	0.0245 (6)	0.0016 (4)	0.0020 (4)	0.0013 (5)
C3A	0.0192 (5)	0.0217 (6)	0.0244 (6)	−0.0005 (4)	0.0029 (4)	0.0049 (5)
C4A	0.0185 (5)	0.0165 (5)	0.0250 (6)	−0.0017 (4)	−0.0003 (4)	0.0042 (4)
C5A	0.0184 (5)	0.0170 (5)	0.0211 (5)	−0.0004 (4)	−0.0003 (4)	0.0012 (4)
C6A	0.0153 (5)	0.0163 (5)	0.0200 (5)	−0.0016 (4)	−0.0012 (4)	0.0032 (4)
C7A	0.0276 (6)	0.0175 (6)	0.0350 (7)	−0.0023 (5)	0.0027 (5)	0.0061 (5)
C8A	0.0183 (5)	0.0153 (5)	0.0204 (6)	0.0008 (4)	0.0000 (4)	0.0021 (4)
C9A	0.0233 (6)	0.0237 (6)	0.0233 (6)	0.0026 (5)	0.0052 (5)	0.0059 (5)
N3	0.0249 (6)	0.0251 (6)	0.0254 (5)	0.0008 (4)	−0.0042 (4)	0.0001 (4)
N4	0.0234 (5)	0.0266 (6)	0.0244 (6)	0.0024 (4)	−0.0054 (4)	0.0024 (5)
C10	0.0180 (5)	0.0231 (6)	0.0210 (5)	0.0005 (4)	0.0003 (4)	0.0049 (4)
C11	0.0175 (5)	0.0257 (6)	0.0213 (6)	0.0004 (4)	−0.0027 (4)	0.0026 (5)
C12	0.0179 (5)	0.0232 (7)	0.0243 (6)	0.0012 (5)	0.0005 (4)	0.0033 (5)
C13	0.0283 (8)	0.0251 (7)	0.0368 (8)	0.0005 (6)	0.0012 (6)	0.0012 (6)
N3A	0.0249 (6)	0.0251 (6)	0.0254 (5)	0.0008 (4)	−0.0042 (4)	0.0001 (4)
N4A	0.0234 (5)	0.0266 (6)	0.0244 (6)	0.0024 (4)	−0.0054 (4)	0.0024 (5)
C10A	0.0180 (5)	0.0231 (6)	0.0210 (5)	0.0005 (4)	0.0003 (4)	0.0049 (4)
C11A	0.0175 (5)	0.0257 (6)	0.0213 (6)	0.0004 (4)	−0.0027 (4)	0.0026 (5)
C12A	0.0179 (5)	0.0232 (7)	0.0243 (6)	0.0012 (5)	0.0005 (4)	0.0033 (5)
C13A	0.0283 (8)	0.0251 (7)	0.0368 (8)	0.0005 (6)	0.0012 (6)	0.0012 (6)

### Geometric parameters (Å, º)

**Table:**

O1—C8	1.2376 (16)	C3A—C4A	1.3900
N1—C1	1.4112 (17)	C3A—C7A	1.508 (3)
N1—H1A	0.9099	C4A—C5A	1.3900
N1—H1B	0.9099	C4A—H4A	0.9500
N2—C8	1.3471 (17)	C5A—C6A	1.3900
N2—C6	1.4347 (17)	C5A—H5A	0.9500
N2—H2A	0.9100	C7A—H7D	0.9800
O1A—C8A	1.238 (3)	C7A—H7E	0.9800
N1A—C1A	1.412 (3)	C7A—H7F	0.9800
N1A—H1C	0.9099	C8A—C9A	1.511 (3)
N1A—H1D	0.9100	C9A—C10A	1.496 (3)
N2A—C8A	1.347 (3)	C9A—H9C	0.9900
N2A—C6A	1.434 (3)	C9A—H9D	0.9900
N2A—H2C	0.8800	N3—C10	1.3425 (18)
C1—C6	1.3995 (19)	N3—N4	1.3635 (19)
C1—C2	1.4022 (19)	N4—C12	1.3534 (19)
C2—C3	1.388 (2)	N4—H4	0.907 (9)
C2—H2	0.9500	C10—C11	1.412 (2)
C3—C4	1.397 (2)	C11—C12	1.3766 (19)
C3—H3	0.9500	C11—H11	0.9500
C4—C5	1.3925 (18)	C12—C13	1.497 (2)
C4—C7	1.5077 (19)	C13—H13A	0.9800
C5—C6	1.3956 (18)	C13—H13B	0.9800
C5—H5	0.9500	C13—H13C	0.9800
C7—H7A	0.9800	N3A—C10A	1.311 (12)
C7—H7B	0.9800	N3A—N4A	1.351 (17)
C7—H7C	0.9800	N4A—C12A	1.331 (16)
C8—C9	1.5119 (18)	N4A—H4B	0.8800
C9—C10	1.496 (2)	C10A—C11A	1.397 (16)
C9—H9A	0.9900	C11A—C12A	1.375 (17)
C9—H9B	0.9900	C11A—H11B	0.9500
C1A—C2A	1.3900	C12A—C13A	1.468 (17)
C1A—C6A	1.3900	C13A—H13D	0.9800
C2A—C3A	1.3900	C13A—H13E	0.9800
C2A—H2B	0.9500	C13A—H13F	0.9800
			
C1—N1—H1A	113.0	C6A—C5A—H5A	120.0
C1—N1—H1B	107.5	C4A—C5A—H5A	120.0
H1A—N1—H1B	111.9	C5A—C6A—C1A	120.0
C8—N2—C6	121.97 (11)	C5A—C6A—N2A	120.1 (13)
C8—N2—H2A	117.4	C1A—C6A—N2A	119.7 (13)
C6—N2—H2A	120.6	C3A—C7A—H7D	109.5
C1A—N1A—H1C	110.0	C3A—C7A—H7E	109.5
C1A—N1A—H1D	113.5	H7D—C7A—H7E	109.5
H1C—N1A—H1D	111.9	C3A—C7A—H7F	109.5
C8A—N2A—C6A	123.6 (15)	H7D—C7A—H7F	109.5
C8A—N2A—H2C	118.2	H7E—C7A—H7F	109.5
C6A—N2A—H2C	118.2	O1A—C8A—N2A	120.4 (18)
C6—C1—C2	118.12 (12)	O1A—C8A—C9A	126 (2)
C6—C1—N1	120.68 (12)	N2A—C8A—C9A	113.7 (11)
C2—C1—N1	121.12 (12)	C10A—C9A—C8A	106.6 (14)
C3—C2—C1	120.77 (13)	C10A—C9A—H9C	110.4
C3—C2—H2	119.6	C8A—C9A—H9C	110.4
C1—C2—H2	119.6	C10A—C9A—H9D	110.4
C2—C3—C4	121.24 (13)	C8A—C9A—H9D	110.4
C2—C3—H3	119.4	H9C—C9A—H9D	108.6
C4—C3—H3	119.4	C10—N3—N4	104.22 (12)
C5—C4—C3	118.03 (12)	C12—N4—N3	112.76 (12)
C5—C4—C7	120.82 (13)	C12—N4—H4	126.7 (16)
C3—C4—C7	121.13 (13)	N3—N4—H4	120.5 (16)
C4—C5—C6	121.19 (12)	N3—C10—C11	111.17 (12)
C4—C5—H5	119.4	N3—C10—C9	119.95 (13)
C6—C5—H5	119.4	C11—C10—C9	128.88 (12)
C5—C6—C1	120.64 (12)	C12—C11—C10	105.37 (12)
C5—C6—N2	119.46 (12)	C12—C11—H11	127.3
C1—C6—N2	119.89 (11)	C10—C11—H11	127.3
C4—C7—H7A	109.5	N4—C12—C11	106.48 (13)
C4—C7—H7B	109.5	N4—C12—C13	122.33 (14)
H7A—C7—H7B	109.5	C11—C12—C13	131.18 (14)
C4—C7—H7C	109.5	C12—C13—H13A	109.5
H7A—C7—H7C	109.5	C12—C13—H13B	109.5
H7B—C7—H7C	109.5	H13A—C13—H13B	109.5
O1—C8—N2	122.20 (12)	C12—C13—H13C	109.5
O1—C8—C9	121.58 (12)	H13A—C13—H13C	109.5
N2—C8—C9	116.22 (11)	H13B—C13—H13C	109.5
C10—C9—C8	111.99 (9)	C10A—N3A—N4A	102.8 (12)
C10—C9—H9A	109.2	C12A—N4A—N3A	117.0 (13)
C8—C9—H9A	109.2	C12A—N4A—H4B	121.5
C10—C9—H9B	109.2	N3A—N4A—H4B	121.5
C8—C9—H9B	109.2	N3A—C10A—C11A	110.3 (14)
H9A—C9—H9B	107.9	N3A—C10A—C9A	125.8 (16)
C2A—C1A—C6A	120.0	C11A—C10A—C9A	123.9 (14)
C2A—C1A—N1A	119.99 (8)	C12A—C11A—C10A	108.4 (13)
C6A—C1A—N1A	120.01 (8)	C12A—C11A—H11B	125.8
C1A—C2A—C3A	120.0	C10A—C11A—H11B	125.8
C1A—C2A—H2B	120.0	N4A—C12A—C11A	101.4 (13)
C3A—C2A—H2B	120.0	N4A—C12A—C13A	127.2 (19)
C4A—C3A—C2A	120.0	C11A—C12A—C13A	131.3 (18)
C4A—C3A—C7A	119.3 (14)	C12A—C13A—H13D	109.5
C2A—C3A—C7A	120.6 (14)	C12A—C13A—H13E	109.5
C3A—C4A—C5A	120.0	H13D—C13A—H13E	109.5
C3A—C4A—H4A	120.0	C12A—C13A—H13F	109.5
C5A—C4A—H4A	120.0	H13D—C13A—H13F	109.5
C6A—C5A—C4A	120.0	H13E—C13A—H13F	109.5
			
C6—C1—C2—C3	−0.18 (19)	C2A—C1A—C6A—N2A	175.7 (15)
N1—C1—C2—C3	−177.18 (12)	N1A—C1A—C6A—N2A	−4.3 (15)
C1—C2—C3—C4	−0.5 (2)	C8A—N2A—C6A—C5A	90 (3)
C2—C3—C4—C5	0.6 (2)	C8A—N2A—C6A—C1A	−86 (3)
C2—C3—C4—C7	−178.08 (13)	C6A—N2A—C8A—O1A	9 (5)
C3—C4—C5—C6	−0.09 (19)	C6A—N2A—C8A—C9A	−173 (2)
C7—C4—C5—C6	178.64 (13)	O1A—C8A—C9A—C10A	59 (4)
C4—C5—C6—C1	−0.60 (19)	N2A—C8A—C9A—C10A	−119 (2)
C4—C5—C6—N2	−179.60 (11)	C10—N3—N4—C12	−0.05 (3)
C2—C1—C6—C5	0.73 (18)	N4—N3—C10—C11	0.05 (2)
N1—C1—C6—C5	177.74 (12)	N4—N3—C10—C9	−179.92 (3)
C2—C1—C6—N2	179.72 (11)	C8—C9—C10—N3	−95.38 (12)
N1—C1—C6—N2	−3.27 (18)	C8—C9—C10—C11	84.65 (12)
C8—N2—C6—C5	90.79 (16)	N3—C10—C11—C12	−0.04 (4)
C8—N2—C6—C1	−88.21 (16)	C9—C10—C11—C12	179.93 (5)
C6—N2—C8—O1	4.1 (2)	N3—N4—C12—C11	0.02 (5)
C6—N2—C8—C9	−176.27 (12)	N3—N4—C12—C13	179.89 (4)
O1—C8—C9—C10	64.49 (19)	C10—C11—C12—N4	0.01 (5)
N2—C8—C9—C10	−115.16 (14)	C10—C11—C12—C13	−179.84 (5)
C6A—C1A—C2A—C3A	0.0	C10A—N3A—N4A—C12A	0.00 (3)
N1A—C1A—C2A—C3A	180.0	N4A—N3A—C10A—C11A	0.00 (3)
C1A—C2A—C3A—C4A	0.0	N4A—N3A—C10A—C9A	180.00 (4)
C1A—C2A—C3A—C7A	177.6 (19)	C8A—C9A—C10A—N3A	−69.6 (15)
C2A—C3A—C4A—C5A	0.0	C8A—C9A—C10A—C11A	110.4 (15)
C7A—C3A—C4A—C5A	−177.6 (18)	N3A—C10A—C11A—C12A	0.00 (5)
C3A—C4A—C5A—C6A	0.0	C9A—C10A—C11A—C12A	179.99 (6)
C4A—C5A—C6A—C1A	0.0	N3A—N4A—C12A—C11A	0.00 (5)
C4A—C5A—C6A—N2A	−175.7 (15)	N3A—N4A—C12A—C13A	180.00 (5)
C2A—C1A—C6A—C5A	0.0	C10A—C11A—C12A—N4A	0.00 (6)
N1A—C1A—C6A—C5A	180.0	C10A—C11A—C12A—C13A	180.00 (6)

### Hydrogen-bond geometry (Å, º)

**Table:**

*D*—H···*A*	*D*—H	H···*A*	*D*···*A*	*D*—H···*A*
N1—H1*B*···O1^i^	0.91	2.13	3.0284 (19)	171
N2—H2*A*···N1^ii^	0.91	2.14	3.0354 (17)	170
C2—H2···O1^i^	0.95	2.62	3.334 (2)	132
N4—H4···O1^iii^	0.91 (1)	1.99 (1)	2.8625 (17)	163 (2)

Symmetry codes: (i) −*x*+1, −*y*+1, −*z*+1; (ii) −*x*+2, −*y*+1, −*z*+1; (iii) −*x*+1, *y*+1/2, −*z*+1/2.
